# Doxorubicin PK/PD modeling in multiple myeloma: towards *in silico* trials

**DOI:** 10.1186/s13062-025-00626-x

**Published:** 2025-03-21

**Authors:** Daniele Andrean, Francesco Da Ros, Mario Mazzucato, Morten Gram Pedersen, Roberto Visentin

**Affiliations:** 1 Department of Information Engineering, University of Padova, Via Giovanni Gradenigo 6b, 35131 Padova, Italy; 2 CRO Aviano, National Cancer Institute, IRCCS, Via Franco Gallini 2, 33081 Aviano, Italy; 3 Clinical Development & Translational Medicine, Aptuit an Evotec Company, Via Alessandro Fleming 4, 37135 Verona, Italy

**Keywords:** Multiple myeloma, PK/PD, Modeling, *in silico*

## Abstract

Doxorubicin (DOXO) is a well-known chemotherapy drug, which is widely used in the treatment of Multiple Myeloma (MM), a treatable but not curable type of blood cancer. Here, we propose a pharmacokinetics and pharmacodynamics (PK/PD) simulation environment, aimed at facilitating the optimization of DOXO treatment regimens in MM treatment. The resulting model has a transparent mechanistic structure, which facilitates its use and interpretation. The simulator was developed using a combination of experimental and modeling techniques, starting from *in vitro* PK/PD experiments conducted on MM cells. In our previous work, we carefully developed a PK model for DOXO in MM cells by fitting experimental data. We now devise a PD model from *in vitro* data investigating the effect of different concentrations of DOXO on cell growth and death in MM cell populations. The PK model is extended to enable a clear mechanistic link between the PK and the PD models, hence providing a complete PK/PD simulator. We show how the mathematical model can be exploited to simulate different DOXO administration protocols with different dosages, repetitions and exposure times, thus, making it possible to explore the effect of a wide range of treatment protocols easily.

## Introduction

Multiple Myeloma (MM) is among the most common types of blood cancer. Depending on the population, it represents 13 to 33% of haematological cancers, affecting 4 out of 100.000 people each year [1]. MM is a treatable but not curable type of cancer, with a 5-year survival rate of $\sim$50% [2]. MM is usually treated with combinations of multiple chemotherapy drugs, of which Doxorubicin (DOXO) is among the most commonly employed and effective ones [3]. Optimizing DOXO treatment regimens is fundamental to enhance therapy efficacy while avoiding the potentially serious side effects of the drug [4].

Specific pharmacokinetics (PK) and pharmacodynamics (PD) studies, at the cellular level, can help in assessing how a certain drug interacts with the cancer cells and provide insight into the effect that a given treatment has on the cellular population. These *in vitro* experiments are preferred to *in vivo* trials as preliminary step, since it is much easier to evaluate both PK and PD at the same time. Moreover, drug resistant cells emerge much more rapidly in *in vitro* conditions due to a higher mutation rate [5], providing crucial information on the effectiveness of the therapy over time. These experiments are however challenging and demanding both in terms of time and materials due to the large number of repetitions that are usually performed [3, 6, 7].

*In silico* trials are increasingly used to complement, or even substitute, *in vitro* experiments [8, 9], since they can readily simulate the response of a biological system to different perturbations. Mathematical modeling has already been successfully applied to study DOXO PK/PD in solid cancers [10, 11]. A mechanistic mathematical model, linking the treatment regimen to the alterations in the targeted cell population, is a fundamental tool to perform *in silico* experiments. Simulations may enable us to explore new therapeutic protocols and elucidate important phenomena involving the action of the drug, while reducing costs and duration of *in vitro* experiments.

We recently developed a mathematical model able to satisfactorily predict DOXO PK following its administration in MM cells [12]. As a natural extension, here we aim at merging the aforementioned model with a new PD model, derived from breast cancer studies [10] and properly modified to specifically describe DOXO effects in MM cells. The resulting PK/PD model links the fast PK dynamics to the slow variations of the cellular populations, and represents a significant step towards the development of a simulator for *in vitro* PK/PD MM experiments.

## Materials and methods

### Pharmacodynamics experiments

All *in vitro* experiments were conducted at Aviano National Cancer Institute, Aviano, Italy. DOXO PD was studied in MM1R cells [13], obtained from American Type Culture Collection (ATCC, http://www.atcc.org). Cells were cultured in Roswell Park Memorial Institute (RPMI) 1640 medium, supplemented with 10% fetal bovine serum (FBS) and penicillin/streptomycin.

MM1R cell cultures were subjected to different DOXO doses to observe the drug effect on vitality and cellular growth. Groups of 5.6$\times$ $10^5$ cells were exposed to 0 (control), 10, 20, 40, 50, 200, 450, or 900 nM DOXO for 3 h. After the exposure, DOXO was removed and, for each concentration, four cell populations were seeded with a density of $10^4$ cells/well in a 96 wells plate. Cells were labeled by applying CFDA-SE 5 $\mu$M for 10 min and the emitted fluorescence was measured with TECAN every 24 h for 14 days, starting after the end of the drug exposure ($t_0=0$). To convert the fluorescence signal of each well into a measure of the number of living cells, a standard curve was created by seeding a specific amount of MM1R cells in 96 wells plats after incubation with CFDA-SE (5 $\mu$M) for 10 min. The emitted fluorescence was then measured with TECAN, and the conversion curve was found by linear regression of fluorescence on cell number. Each type of experiment was initiated on two different days separated by a 3-day interval. This protocol was adopted to reduce the lack of measurements during the weekends or due to experimental failures, and to grant multiple samples (between 4 and 28) for each sampling time. Since each experiment included a control group, free proliferation experiments have $\sim$3 times as many samples than treatment experiments.

### Data processing

All analyses were performed with MATLAB R2022a [14]. Cell counts obtained from the various experiments were grouped according to the administered DOXO concentration and aligned on the same relative timescale to obtain a complete set of repeated measures from $t_0 = 0$ h to $t_{end} = 456$ h for each treatment. Even with repeated experiments starting on different days, some data points were missing because of external causes that prevented measurements (specifically, for 20 and 50 nM at 120 and 288 h).

**Fig. 1:**
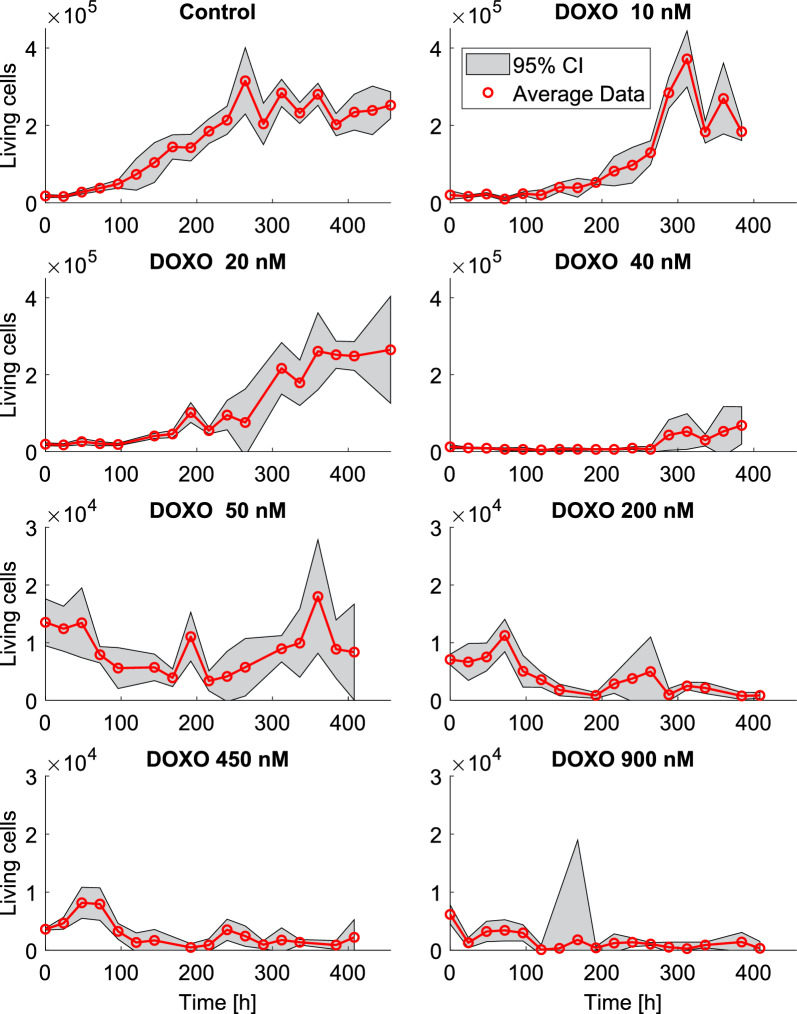
Averaged data grouped by experiment. The 95% CI are represented by the gray areas. Data is presented with two different scales on the *y*-axes to better visualize the data in the experiments with $\ge 50$ nM DOXO

Data points showing unrealistic values, i.e., negative cell counts or cell counts equal to zero before other nonzero measurements, were excluded from the analysis. Such unrealistic values occurred because of relatively low fluorescence signals, which were converted into non-positive cell counts when using the standard curve introduced above. As a consequence, some of the time points resulted with no valid measures, specifically for treatment experiments with DOXO dosed at 200, 450, 900 nM. In total, 10, 20 and 23% data points were excluded for those treatments, respectively. It is worth noting that although percentages might seem high, exluded data points were concentrated on certain sampling times. As a consequence, only 2 time instants remained without data for all the three high DOXO treatments. No imputation methods were used for handling missing data as curves were already well represented and to avoid bias. Mean and standard deviation of the remaining cell counts were calculated at each sampling time, assuming a t-student distribution, to obtain a single cell count value with its confidence interval for each time point. This was performed for each subset of data related to a specific administered DOXO concentration. Average data with their 95% confidence intervals (CI) are reported in Fig. 1.

### PK/PD modeling

Our DOXO PK/PD model is composed of two sub-models, describing DOXO PK and PD, respectively. These are linked by modeling the cell drug-induced death rate (PD parameter) as function of the DNA-bound DOXO concentration (simulated in the PK model). Model equations and parameters are described in detail in the following sections.

#### PK model

**Fig. 2:**
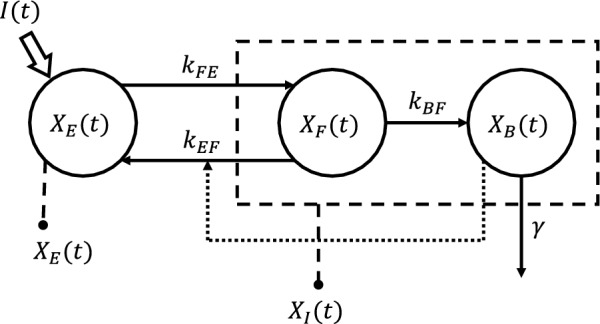
Schematic representation of the PK compartmental model. The dashed rectangle indicates that the free $X_F(t)$ and bound $X_B(t)$ DOXO compartments are measured together as total intracellular DOXO concentration $X_I(t)$

The DOXO PK model adopted in the present work was developed recently by our group [12]. Briefly (see Fig. 2), it consists of a 3-compartment structure describing the extracellular DOXO concentration ($X_E$), the free, intracellular DOXO concentration ($X_F$), and the levels of DNA-bound DOXO ($X_B$). DOXO is administered in the extracellular compartment as described by the function *I*(*t*). After entering the cytoplasm with rate $k_{FE}$, DOXO can either irreversibly bind to the DNA with rate $k_{BF}$ or leave the cell, returning to the extracellular space with rate $k_{EF}$ that depends nonlinearly on $X_B$. The nonlinearity in $k_{EF}$ reflects the fact that overexpression of P-glycoprotein, a transmembrane efflux pump involved in pharmacoresistance, is dependent upon DOXO concentrations [15].

At difference with [12], here we assumed that DOXO loses its efficacy over time due to dilution in the cells’ cytoplasm following replication and because of degradation of the drug. This is supported by experimental data, which sometimes exhibit a cellular regrowth after low-dose treatments (Fig. 1). This phenomenon has been modeled by adding an exponential decay factor $\gamma$ describing the DNA-bound DOXO kinetics, and enabling a decrease in cellular death rate over time. The complete set of model equations is the following:

1 $$\begin{aligned} {\left\{ \begin{array}{ll} \frac{dX_E(t)}{dt} = k_{EF}(X_B(t))\frac{V_I}{V_E}X_F(t) - k_{FE}X_E(t) + I(t)\ , &  X_E(0)=I(0)\\ \frac{dX_F(t)}{dt} = k_{FE}\frac{V_E}{V_I}X_E(t) - k_{EF}(X_B(t))X_F(t) - k_{BF}X_{{F}}(t)\ , &  X_F(0)=0\\ \frac{dX_B(t)}{dt} = k_{BF}X_F(t)-\gamma X_B(t)\ , &  X_B(0)=0 \end{array}\right. } \end{aligned}$$

with

2 $$\begin{aligned} k_{EF}(X_B(t)) = \frac{V_{max}X_B(t)^{1.31}}{k_{th}^{2.31} + X_B(t)^{2.31}}. \end{aligned}$$

Values of PK parameters appearing in Eqs. (1) and (2) were taken from [12] and are reported in table 1.

**Table 1:** PK model parameters estimated in [12]

Parameter	Value	Unit
$V_{max}$	1.65$\cdot$ $10^{4}$	nM/h
$k_{th}$	464	nM
$k_{FE}$	5.63$\cdot$ $10^{-4}$	1/h
$K_{BF}$	1.22	1/h
$V_E$	100	$\mu$L
$V_I$	0.07	$\mu$L

#### PD model

The PD model consists of a logistic function describing the number of living cells over time. In a population initially having $N_0$ cells, the variation of cell counts in time *N*(*t*) is described by a single differential equation that takes into account the balance between proliferation rate $k_p$, drug-induced death rate $k_d$, and the capacity of the culture wells $\theta$:

3 $$\begin{aligned} \frac{dN(t)}{dt} = k_p\, N(t) \left( 1-\frac{N(t)}{\theta }\right) -k_d\big (X_B(t)\big )\, N(t) , \quad \quad N(0)=N_0. \end{aligned}$$

DOXO’s main action is to cause DNA damage, disrupting cellular replication and inducing death by apoptosis [4]. Since the experimental data (Fig. 1) show that the biggest differences in the population’s evolution occur between the low-DOXO experiments (DOXO $\le$ 50 nM) rather than among high-DOXO experiments, we assumed a maximum rate-of-damage that DOXO can induce on DNA. Consequently, the death rate is modeled as a saturating Michaelis-Menten function of DNA-bound DOXO,

4 $$\begin{aligned} k_d\big (X_B(t)\big )=K_{dmax}\frac{X_B(t)}{X_{BHS}+X_B(t)}\ . \end{aligned}$$

### Model identification

All the following operations have been implemented in MATLAB and model parameters were estimated using nonlinear least-squares estimation provided by the *lsqnonlin()* function. In particular, almost all the PK model parameters have been fixed to their respective values estimated in the previous work [12], while PD model parameters ($k_p$, $K_{dmax}$, $X_{BHS}$, $\theta$) and the exponential decay ($\gamma$) were estimated by identifying the entire model on DOXO PD data. Structural identifiability analysis was performed using the DAISY software [16] to assess *a priori* identifiability of the full PK/PD model. Assuming the PK parameters as known, the model resulted *a priori* globally identifiable. Indeed, the addition of the the decay term and the introduction of the PD subsystem do not alter the dynamics of the PK subsystem. Therefore, the PK model can be identified as a stand-alone model on PK data only [12].

It is worth noting that the first PD data sample is highly unreliable due to experimental issues. In fact, in many occasions the first sample was higher than the second one, even in control experiments where the number of cells should only increase due to the absence of DOXO (see Fig. 1). Rather than discarding the first data point or using it as initial condition, we allowed the model to fit this data point with some degree of freedom. Thus, the initial cell count $N_0$ was considered as an additional unknown parameter, and the initial data point was considered as prior for its estimation. Notably, this addition did not compromise the global identifiability of the model. Hence, the vector of model parameters to be estimated was

5 $$\begin{aligned} \textbf{p}= \{k_p, K_{dmax}, X_{BHS}, \theta , \gamma , N_0\}. \end{aligned}$$

Each parameter was assumed to have the same value across experiments, except $N_0$, which was allowed to vary between experiments. As a consequence, we performed simultaneous model identification on all the average data points of each treatment regimen, with control data weighted 3 times more than treatment data. This weighting was done to obtain reliable estimates of $k_p$ and $\theta$, which are unaffected by the presence of DOXO, and are therefore favorably found from, mainly, the control data.

The PK/PD model was identified using a Maximum a Posteriori (MAP) estimator [17]. In particular, the implemented MAP cost function $L$ is an extension of the cost function for Least Squares that accounts for prior knowledge on the unknown parameters, if available,

6 $$\begin{aligned} L (\textbf{p})=(\textbf{y}-{\mathbf {\hat{y}(p)}}) \Sigma _v^{-1} (\textbf{y}-{\mathbf {\hat{y}(p)}}) + (\mathbf {\mu _{p}}-\textbf{p}) \Sigma _p^{-1} (\mathbf {\mu _{p}}-\textbf{p}). \end{aligned}$$

This function of the parameter vector $\textbf{p}$ is composed of two terms: the first addend accounts for the difference between data $\textbf{y}$ and model prediction ${\mathbf {\hat{y}(p)}}$, weighted by data covariance $\Sigma _v^{-1}$; the second term considers a penalty proportional to the difference between the expected parameters values $\mathbf {\mu _{p}}$ and the estimated parameters $\textbf{p}$, weighted by the uncertainty on the expected values $\Sigma _p^{-1}$. Therefore, the MAP estimation realizes the best trade-off between model adherence to experimental measures and previous knowledge on model parameters.

In particular, *a priori* information was considered for $k_p$ assuming a doubling time of 72 h for MM1R cells (i.e., 0.028 h^-1^, http://www.atcc.org), for $\theta$ assuming a maximum carrying capacity of $2.5\cdot 10^5$ cells (determined by prior experiments), and for $N_0$ using the cell count measured at the beginning of each experiment. No prior was assumed on drug-induced death rate parameters $K_{dmax}$, $X_{BHS}$ and the decay parameter $\gamma$. In addition, for all model identifications the 95% CI of model prediction was determined using a bootstrap approach on data. Specifically, for each experiment, the average data point was randomly replaced by one of the single measurements used to calculate it. Model identification was then performed on this new dataset and results were stored. The 97.5% and the 2.5% percentiles of all the model predictions were used as upper and lower confidence bounds, respectively. This bootstrap approach was also used to obtain standard errors on parameter estimates.

## Results

### Model fit and estimated parameters

**Fig. 3:**
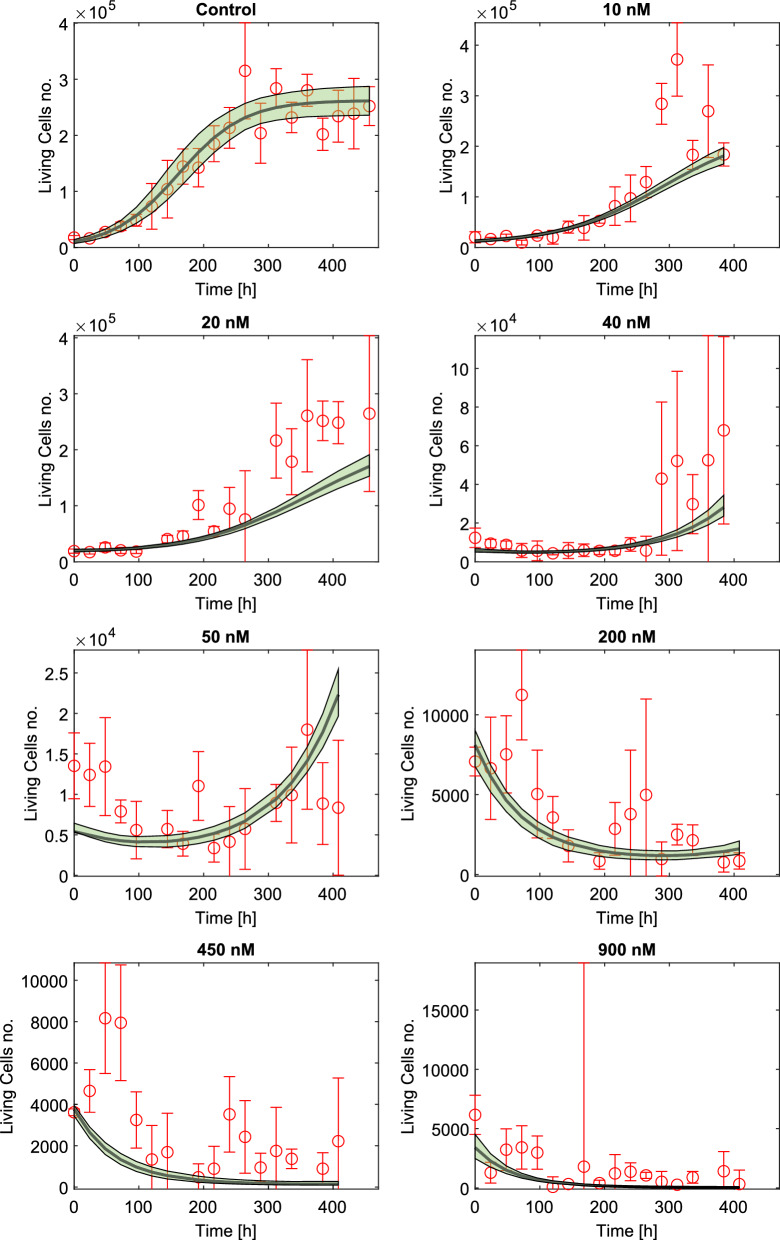
Model identification results on all data. Data points are reported with their 95% CI (red bars). The resulting fit (black line) is plotted together with its 95% CI (green area), calculated with a bootstrap approach. Note that, to improve visualization, different scales have been used on the *y*-axes in the panels

Results of model fits are reported in Fig. 3. Note that, for the sake of readability, there is a considerable difference in the scales that were adopted for each experiment.

**Table 2:** Estimated values for model parameters with their standard deviation (SD) obtained by bootstrapping, and the corresponding coefficients of variation (CV)

Parameter	Unit	Estimate	SD	CV %
$k_p$	1/h	0.0198	0.002	10.3
$\theta$	dimensionless	262209	13686	5.2
$K_{dmax}$	1/h	0.0435	0.0025	5.7
$X_{BHS}$	nM	47.5	9.83	20.7
$\gamma$	1/h	0.0044	0.0004	10.3
$N_0$ (ctrl)	dimensionless	10370	2043	19.7
$N_0$ (10nM)	dimensionless	12080	1140	9.4
$N_0$ (20nM)	dimensionless	19586	1224	6.2
$N_0$ (40nM)	dimensionless	5596	530	9.4
$N_0$ (50nM)	dimensionless	5411	274	5.1
$N_0$ (200nM)	dimensionless	8101	611	7.5
$N_0$ (450nM)	dimensionless	3785	151	4.0
$N_0$ (900nM)	dimensionless	3394	575	16.9

The estimated model parameters are reported in Table 2 with their variability and precision expressed by standard deviation (SD) and coefficients of variation (CV), respectively. Parameter estimates are precise, as indicated by the low CVs, and robust to slight changes in data, as proved by the narrow confidence intervals obtained from the bootstrapping operation (Fig. 3).

The estimated values of the proliferation parameters ($k_p$ and $\theta$) are sensible, as indicated by the good fit to the control group with narrow confidence intervals (Fig. 3). Moreover, the estimated $k_p$ value is in reasonable agreement with the one indicated in the MM1R documentation (ATCC, http://www.atcc.org). The estimate of the carrying capacity $\theta$ is also trustworthy, since it is close to the maximum carrying capacity prior. Thus, we conclude that these parameters are faithfully estimated. Parameters related to the effect of the drug ($K_{dmax}$, $X_{BHS}$) are also reliably estimated and correctly induce delays in cellular growth or population suppression, depending on the treatment (Fig. 3). The decay term $\gamma$ enables the simulated number of cells to regrow after the low DOXO treatments ($\le$ 50 nM), as suggested by treatment data in Fig. 3.

As expected, estimates of the initial number of cells do not agree perfectly with some of the initial point measurements. Since some of the initial measurements were highly unreliable and implausible (e.g., higher than the second measure in the control experiments), we believe it is more robust to estimate the initial values rather than using the first data point. Indeed, estimating initial points leads to better overall model fits.

### Model validation

**Fig. 4:**
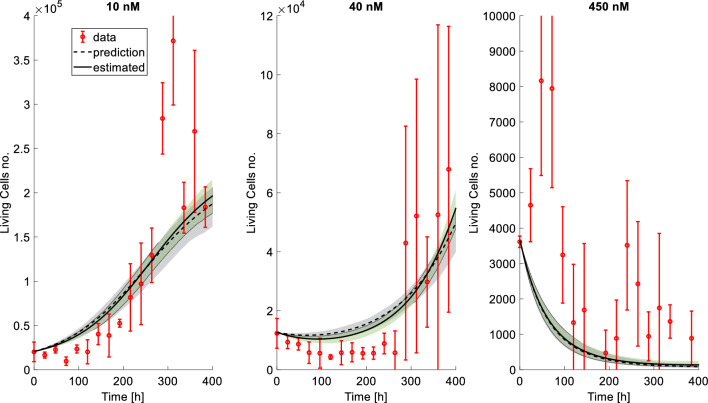
Simulated treatments with the model trained on all data (estimated) and on validation data (prediction). Data points are reported with their 95% CI (red bars). Predictions with the validation model and simulations with the full model are plotted together with their 95% CI (solid lines with green areas and dashed lines with gray areas respectively), calculated with a bootstrap approach. Each simulation started from the initial data point of each treatment. Note that, to improve visualization, different scales have been used on the *y*-axes in the panels

To verify that the developed model is useful for simulating unseen scenarios, we tested the ability of our model to predict data that has not been used to estimate the model parameters. We fitted the model to a reduced version of the dataset with only control, 20 nM, 50 nM, 200 nM and 900 nM traces. Compared to the ones obtained using the entire dataset (Table 2), parameter estimates slightly differ but are within ±3 SD ($k_p=0.0179\pm 0.0013$ 1/h, $\theta =259710 \pm 7375$, $K_{dmax}=0.046\pm 0.0047$ 1/h, $X_{BHS}=74.4 \pm 22.8$ nM, $\gamma = 0.0033 \pm 0.0007$ 1/h). Subsequently, we simulated the scenarios that were not used to estimate the parameters (10 nM, 40 nM and 450 nM) to understand whether our model with the obtained parameters could correctly predict data trends for these unseen cases. We used the first (experimentally observed) data point as initial point of the simulations, assuming that a user would only have access to that measure at the beginning of an experiment, and found that the model with parameters estimated from the reduced dataset can correctly predict unseen data (Fig. 4). For comparison, we also simulated the traces for these three doses with the parameters obtained by fitting the model on the whole dataset (Table 2), starting from the initial data point. The simulated cellular growth curves, obtained with the parameters identified either on the full or on the reduced datasets, were almost identical (Fig. 4). Based on these results, we conclude that the model is robust and can be used to simulate treatment regimens not used for model identification.

### PK/PD simulation

The developed PK/PD model with the estimated parameters can be used to simulate and evaluate different DOXO treatment regimens. As an illustrative case, we simulated 50 h of free cellular proliferation starting from $N_0$ = 100,000 cells, followed by three different DOXO treatment regimens:

(a) a single 200 nM DOXO bolus, administered at $t = 50$ h, removed after 3 h;

(b) two 100 nM DOXO boluses, administered at $t = 50$ h and $t = 350$ h, each one removed after 3 h;

(c) four 50 nM DOXO boluses, administered at $t = 50, 150, 300, 450$ h, each one removed after 3 h.

**Fig. 5:**
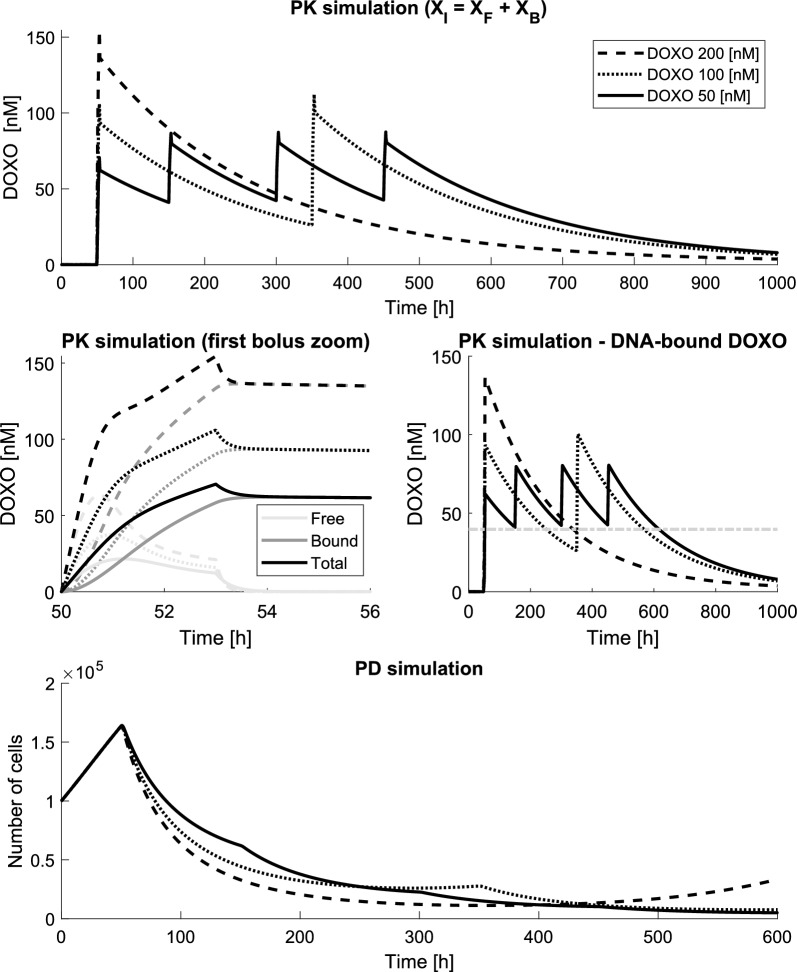
Example of PK/PD simulation. 3 experiments were simulated to compare the efficacy of single bolus high DOXO treatments vs a multi-bolus low DOXO treatment. The upper panels shows the total intracellular DOXO, sum of the DNA-bound and the cytosolic DOXO. The middle panes show a zoom around the first DOXO bolus and the DNA-bound DOXO over time respectively. The lower panel reports the PD simulation results with the number of living simulated cells over time

The simulated results are shown in Fig. 5. The upper three panels show the PK response of the model to the treatments. In particular, the horizontal line in the graph showing DNA-bound DOXO ($X_B$) represents the efficacy concentration threshold, i.e., the minimum amount of DOXO required to trigger cellular death, which can be found as the value of $X_B$ by which $k_d$ exceeds $k_p$. The 50 nM treatment was designed to maintain the level of DNA-bound DOXO above the threshold for the longest time using four low-DOXO boluses. Indeed, this “optimized” treatment shows excellent tumor reduction performance as it never allows cellular regrowth (Fig. 5, lower panel). The protocol composed of two 100 nM boluses has similar performance but leads to two high DOXO peaks, which would cause increased risks for the patient, since maximum DNA-bound DOXO concentration is linked to the instantaneous toxicity effect that the drug exerts on both tumor and healthy cells. The 200 nM treatment has the worst performance with respect to inducing cell death, exhibiting substantial cellular regrowth. Moreover, it also reaches the highest DOXO concentrations, and therefore the highest risk of side effects.

## Discussion and conclusions

Optimization of Multiple Myeloma therapy is a complex and ongoing research field. We addressed one of its basic aspects, i.e., *in vitro* experiments, with the aim of devising a proper mechanistic model linking a specific DOXO-based MM treatment to its effects on the dynamics of the cell population size. Our proposed solution represents the first step towards a complete PK/PD model, validated to perform *in silico* experiments, and consequently accelerating therapy optimization research aimed at maximizing treatment efficacy while minimizing possible side effects.

Adding the PD model to our PK model [12] offers a more complete and powerful tool to link a specific treatment regimen, expressed in terms of administered DOXO doses, to the number of living cells in the culture well. The white-box nature of our PK/PD simulator also means that it is more interpretable by providing more direct links to the biochemical mechanisms that the drug leverages to induce cellular death. Moreover, the mechanistic nature of the model ensures that specific physiological constraints are respected by the simulated responses.

The validation step confirms that our model can correctly describe cellular evolution even in new, unseen experiments. Given the almost perfect agreement between the simulations (Fig. 4), we propose the parameters derived from the full dataset (Table 2) as the optimal choice, as they exhibit lower uncertainty and are derived using all the information in our experimental dataset.

We illustrated the utility of the PK/PD model by simulating a simple case study, from which important information about DOXO administration protocols can be extracted. Although our results would need proper validation with further *in vitro* experiments, we believe that *in silico* trials are a valuable tool that could boost pharmacological research and help in delivering new therapeutic protocols faster.

However, some aspects have to be taken into account. First, we assumed that the DNA-bound DOXO decreases over time to account for the drug’s loss of efficacy. This may be not true in reality as we lack PK experiments over a long time span. Such experiments are particularly challenging to perform as, after each cellular replication, the intracellular DOXO in the newborn cells will be halved, leading to a progressively weaker fluorescence signal. We also did not collect genomics data that could indicate the presence of drug-resistant cells, which could outnumber the the dying drug-sensitive cells and overtake the population dynamics [18].

The 50 nM experiment generated perplexing results; some of the single experiments seemed to show cellular regrowth towards the end of the experiment while others showed suppression of the population. This suggests that the cell populations are heterogeneous, a feature that could be incorporated in future versions of the model to increase its ability to accurately capture the dynamics near the half-max value for the death-rate function, which we estimated to be $X_{BHS}\sim 47.5$ nM. Moreover, longer PD experiments would be needed to better understand these specific treatment conditions. However, cultivating cells for such a long time may lead to greater influence of confounding factors such as nutrient availability; refreshment of the culture media caused fluctuations in the living cells number, contributing to discrepancies between model simulations and experimental data. This operation was consistently carried out around $t=300$ h for all the low DOXO dosages (DOXO $\le 50$ nM). As it clearly appears from Figs. 1 and 3, the largest data outliers lie around $t=300$ h and likely correspond to refreshment-induced proliferation. Currently, our model does not account for nutrient availability and, therefore, it ignores these abrupt increase in the number of cells.

High DOXO treatments (DOXO $\ge 200$ nM) present an initial rise in cell number, followed by a steady fall that stabilizes at low values, likely indicating the suppression of the population. This early fluctuation may be caused by the initial plate rinsing to remove DOXO, which includes a complete medium renewal, thus providing the cells with the maximum concentration of nutrients. In this regard, we ignored this initial trend to avoid designing too complex models, considering that the number of cells involved in this phenomenon is limited (around $10^4$) compared to the carrying capacity of the wells (around 2.5$\times$ $10^5$). Moreover, the model is still able to capture the population decline that takes place subsequently (Fig. 3).

While it is true that the estimation of the initial points enabled us to ignore unreliable measures and obtain better fits, we have to consider that this strategy led to poor fitting of plausible data in the 40 nM and 50 nM DOXO experiments (Fig. 3). This effect can be explained by considering that our model is not meant to fit initial fluctuations in cell number, e.g., due to delays in the drug’s action or the previously mentioned plate rinsing. Rather, we focused our efforts towards the description of long-term behaviors in the cellular population. Considering the traces where the magnitude of initial fluctuations is comparable with the maximum value recorded (i.e., experiments with DOXO $\ge$ 50 nM), the associated cost function is not dominated by errors on the last data points ($t \ge 200$ h), in contrast to what happens in the experiments where the carrying capacity is reached. Therefore, the $N_0$ estimates tend to over-compensate for initial errors, caused by unexplained phenomena.

Our work can be further enhanced by collecting data on other MM cells lines and integrating their sets of parameters in the model. This would enable us to simulate the effect of a single treatment on many different MM cells populations, providing a more complete and exhaustive picture of treatment effects.

## Data Availability

The dataset supporting the conclusions of this article is available in the ‘Research Data UNIPD’ repository, https://researchdata.cab.unipd.it/1451.
